# Person-Centered Health Care: An Approach That Integrates Acute and Long-Term Care

**DOI:** 10.1093/geroni/igaa057.2479

**Published:** 2020-12-16

**Authors:** Yuchi Young, Ye-Mei Chen, Kuo-Piao Chung, Patrick Schumacher, Hsiu-Hsi Chen, Yen-Po Yeh

**Affiliations:** 1 SUNY at Albany, Rensselaer, New York, United States; 2 National Taiwan University, Taipei, Taiwan (Republic of China); 3 Institute of Health Policy and Management, National Taiwan University, Taipei, Taipei, Taiwan; 4 University at Albany State University of New York, Rensselaer, New York, United States; 5 Changhua County Public Health Bureau, Changhua, Taiwan (Republic of China)

## Abstract

The concept of person-centered health care (PCHC) shifts from the individual’s diagnosis to the holistic health and individual social needs. PCHC is defined by WHO as “empowering people to take charge of their own health rather than being passive recipients of services.” This care strategy is based on the belief that if an individual is an equal partner in care planning and care decision making process, and that his/her view, input, and experiences can help improve overall health outcomes. PCHC requires a reconfiguration of the health care system. A vertical or/and horizontal integration of a variety of healthcare settings is necessary. A proposed 5-year PCHC pilot study in Taiwan’s Changhua county aims to reduce unnecessary hospitalizations by 15% in 4 years. Year one will focus on alignment of health care providers, baseline data collection, and assessment plans development. PCHC intervention will start in years two to five. Part of a symposium sponsored by International Comparisons of Healthy Aging Interest Group.

